# Presence of Pleural Effusion in the Assessment of Remote Dielectric Sensing

**DOI:** 10.3390/jcm12134415

**Published:** 2023-06-30

**Authors:** Teruhiko Imamura, Toshihide Izumida, Riona Yamamoto, Yu Nomoto, Kousuke Aakao, Koichiro Kinugawa

**Affiliations:** Second Department of Internal Medicine, University of Toyama, 2630 Sugitani Toyama, Toyama 930-0194, Japanapollon080607@gmail.com (K.A.);

**Keywords:** cardiology, pulmonary edema, monitoring

## Abstract

Background: The remote dielectric sensing (ReDS) system is a recently introduced non-invasive technology used to easily estimate the degree of lung fluid volume without any expert techniques. In the previous literature, ReDS values had a moderate correlation with invasively measured pulmonary artery wedge pressure (PAWP), the gold standard for representing left heart preload. Considering the mechanism of ReDS technology, ReDS values may be inappropriately elevated in the presence of pleural effusion (PE), and the ability of the ReDS system to estimate PAWP may decrease in such a situation. Methods: In-hospital patients with cardiovascular diseases underwent computed tomography, and the presence of pleural effusion (PE) was evaluated. The measurement of ReDS values using the ReDS system and the measurement of PAWP using invasive right heart catheterization were also performed simultaneously. The impact of the presence of PE on the relationship between the ReDS values and PAWP was evaluated. Results: A total of 59 patients (79 years, 30 male) were included. The median ReDS value was 25% (IQR: 22%, 30%) and the median PAWP level was 13 (IQR: 10, 18) mmHg. Of these patients, 11 had PE. PAWP was not significantly different between the two groups, whereas the ReDS values in the PE group were significantly higher than in the no-PE group. The impact of the presence of PE on the ReDS values was significant, with a beta value of 6.61 (95% confidence interval: 4.80–8.42, *p* < 0.001) upon adjusting for the levels of PAWP. Conclusions: We should pay attention to interpreting ReDS values when assessing the degree of pulmonary congestion in patients with PE, because ReDS values may be inappropriately elevated in this cohort.

## 1. Introduction

Accurate assessment of the degree of pulmonary congestion is key to the successful management of congestive heart failure and to improving clinical outcomes in patients with congestive heart failure by adjusting diuretic therapy [1]. The detection of occult pulmonary congestion and early intervention with such a mild pulmonary congestion are of great importance to avoid the development of clinically obvious congestive heart failure, even in less sick patients without any signs of heart failure [2].

Several clinical modalities are used to assess pulmonary congestion, including chest X-ray, plasma B-type natriuretic peptide levels, lung ultrasound, computed tomography, and invasive right heart catheterization [3]. Of these, right heart catheterization is the gold standard for quantifying the degree of pulmonary congestion [4]. However, it is invasive, painful, and sometimes triggers hemodynamic deterioration, especially in patients with unstable hemodynamics. Thus, in real-world clinical practice, these modalities are used in combination, depending on the clinical scenario.

The remote dielectric sensing (ReDS) system is a new technology that non-invasively estimates lung fluid volume based on electromagnetic energy and displays a percentage within one minute on a monitor [5]. Its ability has been validated in comparison with other modalities, including computed tomography, right heart catheterization, chest X-ray, lung ultrasound, and plasma B-type natriuretic peptide levels [6,7,8,9,10,11,12]. ReDS is approved by the FDA and CE for lung fluid monitoring, as well as in other countries, including Japan. ReDS is clinically used in various clinical settings such as out-patient clinics, emergency departments, and in-hospital management [5].

Several factors appear to influence ReDS values. ReDS values are overestimated in patients with chronic pulmonary obstructive disease [13]. Obesity with excessive extra-thoracic fat tissue is also associated with overestimated ReDS values. However, the impact of the presence of pleural effusion (PE) on ReDS assessment remains unknown. PE is sometimes complicated in patients with a variety of diseases, including heart failure [14]. We theoretically hypothesized that the presence of PE may be associated with inappropriately elevated ReDS values and could result in an inaccurate assessment of pulmonary congestion. In this study, we investigated the association between the presence of PE and the accuracy of ReDS values in estimating pulmonary artery wedge pressure (PAWP), which is a surrogate for pulmonary congestion.

## 2. Methods

### 2.1. Study Design

In this cross-sectional observational study, patients who had undergone the following procedures were included: chest computed tomography, ReDS measurements, and right heart catheterization. The impact of the presence of PE on the difference between ReDS values and PAWP was evaluated.

### 2.2. Patient Selection

**Figure 1:**
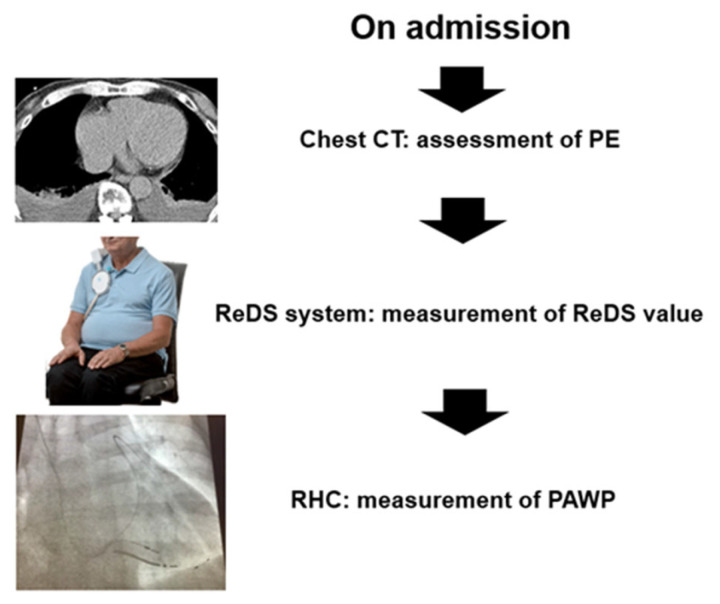
Study design. After the admission, chest CT, ReDS measurement, and RHC were performed simultaneously in patients with cardiovascular diseases. CT, computed tomography; PE, pleural effusion; ReDS, remote dielectric sensing; RHC, right heart catheterization; PAWP, pulmonary artery wedge pressure.

Hospitalized patients with cardiovascular diseases who had undergone the following three tests simultaneously in a clinically stable situation were retrospectively included in this study: (1) ReDS measurements, (2) right heart catheterization, and (3) chest computed tomography (Figure 1). Patients who had been admitted for acute coronary syndrome were excluded. Patients with active infections, such as sepsis, did not undergo right heart catheterization and were not included. Patients with physical abnormalities that were inappropriate for ReDS devices and those with body mass indexes below 15.0 or above 35.0 were excluded. All patients provided written informed consent upon admission. This study was approved by local ethics committee (MTK2020007, 3 March 2021).

### 2.3. ReDS Measurement

**Figure 2:**
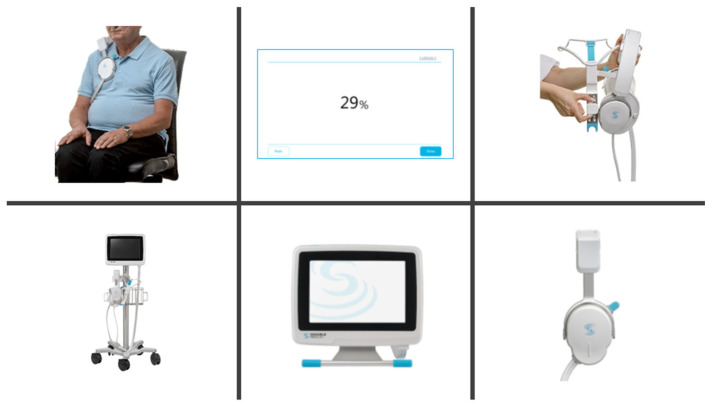
Sensor units and monitor for remote dielectric sensing. The patient wears the sensor units on the right shoulder. The patient is asked to sit and perform natural breathing. After one minute, ReDS value is displayed on the monitor. ReDS value represents the degree of lung fluid volume.

The ReDS system is a recently developed non-invasive electromagnetic-energy-based technology used to quantify lung fluid levels within a minute of application (Figure 2). ReDS employs low-power electromagnetic signals emitted between the two sensors embedded in a wearable device. The analyzed signal reflects the dielectric properties of the lung portion between the sensors.

ReDS values were measured in 45–60 s with the patients in a seated resting position, breathing normally, using two sonar sensors worn on the right shoulder [15]. The displayed ReDS values represented the estimated percentage of lung fluid volume. The manufacturer-recommended normal range of ReDS values is between 20% and 34%, although detailed validation studies in real-world clinical settings have not yet been conducted [5].

### 2.4. Right Heart Catheterization

Invasive right heart catheterization was performed via the right jugular vein under local anesthesia to directly measure intra-cardiac pressure and estimate cardiac function by board-certified cardiologists in a specialized catheter laboratory. Hemodynamic parameters were measured in a standard manner during breath-holding in an expiratory state, including right atrial pressure, pulmonary artery pressure, PAWP, and cardiac output. Raw data were double-checked and a consensus was established by multiple cardiologists who were blinded to the measured ReDS values in each patient.

### 2.5. Chest Computed Tomography

**Figure 3:**
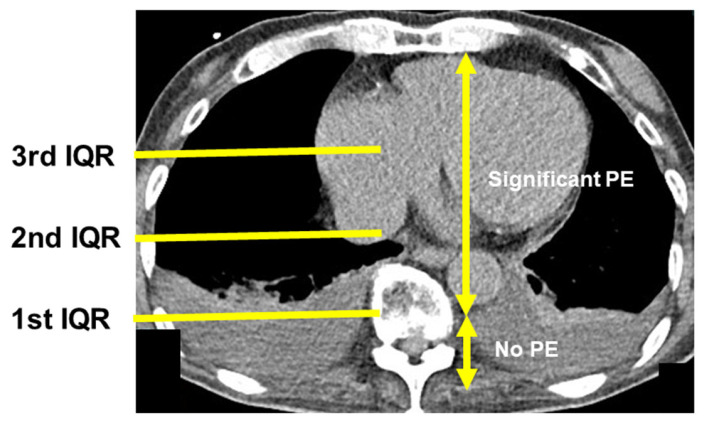
How to semi-quantify the degree of pleural effusion using chest computed tomography. An anteroposterior quartile of the right lung was used to define the degree of pleural effusion. The anteroposterior quartile was defined as the most anterior quartile in which the pleural effusion was observed on the axial image above the ipsilateral hemidiaphragm. In this study, effusion extending beyond the first anteroposterior quartile was defined as significant pleural effusion. IQR, interquartile range; PE, pleural effusion.

Chest computed tomography images without enhancement were obtained upon admission to semi-quantify the degree of right thoracic PE. An anteroposterior quartile was used to define the degree of PE by a researcher (TI) who was blinded to the results of the ReDS assessment and right heart catheterization at the time of measurement [16]. The anteroposterior quartile was defined as the most anterior quartile, in which the PE was observed on the axial image above the ipsilateral hemidiaphragm. The PE had the greatest thickness at this slice. Effusion extending beyond the first anteroposterior quartile was defined as significant PE (see Figure 3 as an example of significant PE).

### 2.6. Statistical Analysis

Continuous variables were expressed as median and interquartile range (IQR) and compared between the two groups using a Mann–Whitney U test. The normality of their distribution was confirmed using the Shapiro–Wilk test. Categorical variables were expressed as numbers and percentages and compared between the two groups using Fischer’s exact test. The correlation between ReDS values and PAWP was evaluated using Pearson’s correlation coefficient. Linear regression analysis was performed to assess the relationship between ReDS values and PAWP following confirmation of the normality of residuals. The impact of the presence of PE on ReDS values was evaluated via analysis of covariance, in which PAWP was used as a covariant. Statistical analyses were performed using SPSS Statistics 26 (IBM, Armonk, NY, USA). Two-sided *p*-values < 0.05 were considered statistically significant.

## 3. Results

### 3.1. Baseline Characteristics

**Figure 4:**
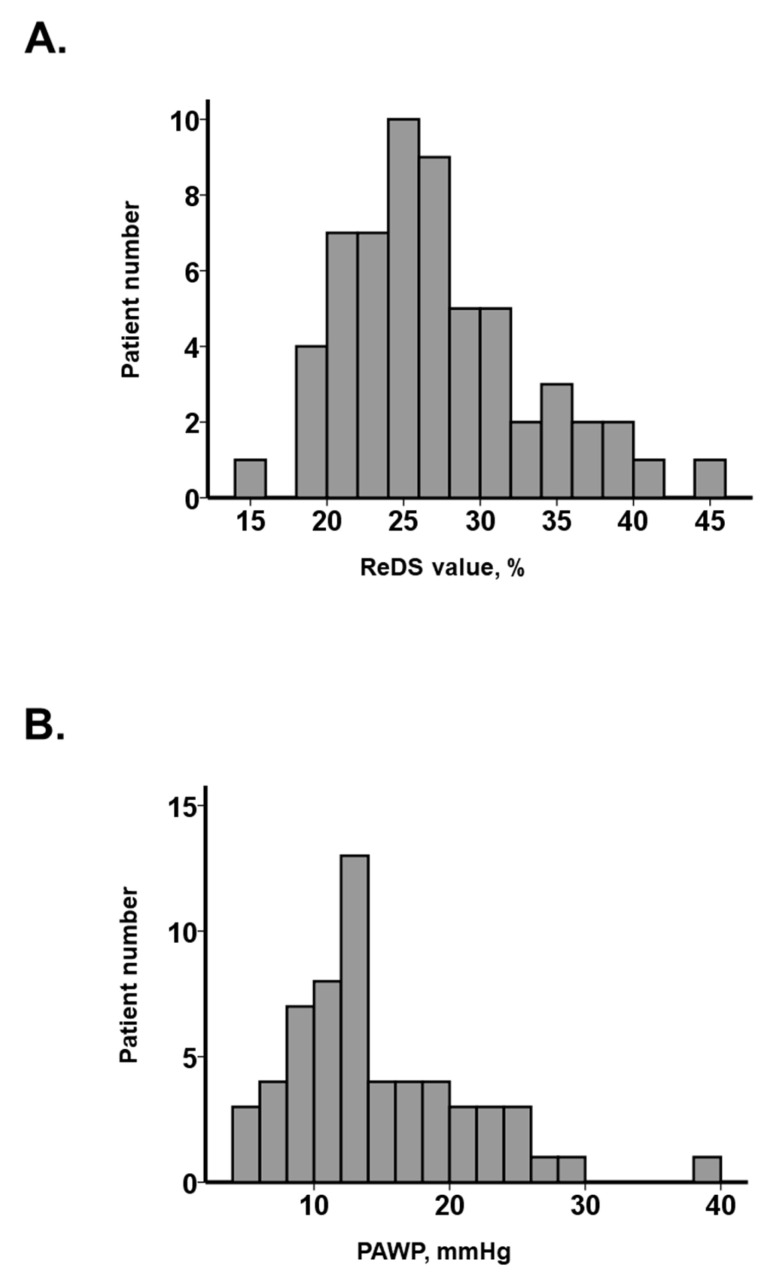
Distribution of ReDS values (**A**) and PAWP (**B**). ReDS, remote dielectric sensing; PAWP, pulmonary artery wedge pressure.

A total of 59 patients were included (Table 1). The median age was 79 (IQR: 72, 84) years and 30 (51%) patients were male. Half of the patients (53%) had experienced heart failure. The median left ventricular ejection fraction was 54% (IQR: 40%, 69%). The median plasma B-type natriuretic peptide level was 231 (IQR: 82, 474) pg/mL. No patients had significant lung diseases, including pulmonary pneumonia, lung cancer, and chronic obstructive pulmonary disease. The ReDS values and PAWP were distributed widely, with median values of 25% (IQR: 22%, 30%) and 13 (IQR: 10, 18) mmHg, respectively (Figure 4A,B).

### 3.2. Presence of PE

A total of 11 patients had PE between the first and second interquartile lines, indicating the presence of significant PE (PE group). No patients had PE greater than the second interquartile line (Figure 3). The other 48 patients had no PE or PE below the first interquartile line, indicating no significant PE (no-PE group). Patients in the PE group had higher plasma B-type natriuretic peptide levels and ReDS values (*p* < 0.05 for both; Table 1). Most of the other variables were not significantly different between the two groups (*p* > 0.05).

### 3.3. Impact of PE on the Relationship between ReDS Values and PAWP

**Figure 5:**
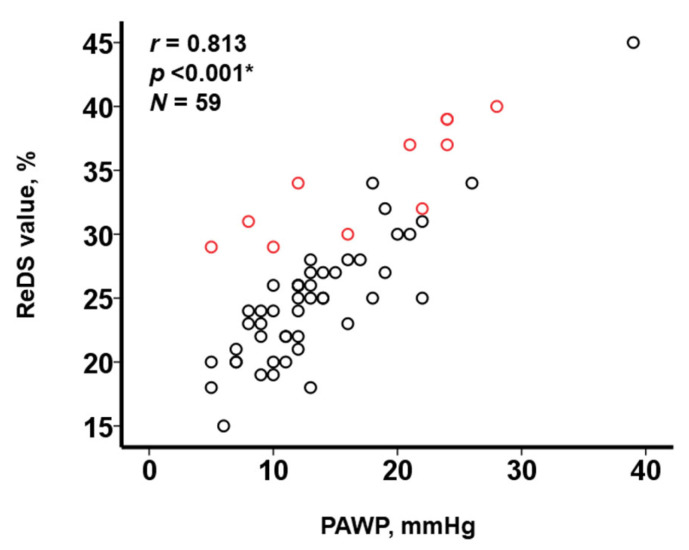
Relationship between ReDS values and PAWP. ReDS, remote dielectric sensing; PAWP, pulmonary artery wedge pressure. Red dots represent PE group and black dots represent no-PE group. ReDS values and PAWP have a moderate correlation (* *p* < 0.05 according to Pearson’s correlation coefficient). More red dots are located in upper region than the black dots with similar trends.

The ReDS values and PAWP had a moderate correlation in all cohorts (r = 0.813, *p* < 0.001, *N* = 59; Figure 5). Here, more dots of the PE group (red dots) were distributed in the upper region than those of no-PE group (black dots). For example, in patients with PAWP around 10 mmHg, ReDS values were distributed between 20% and 25% in the no-PE group, whereas ReDS values were distributed around 30% in the PE group.

**Figure 6:**
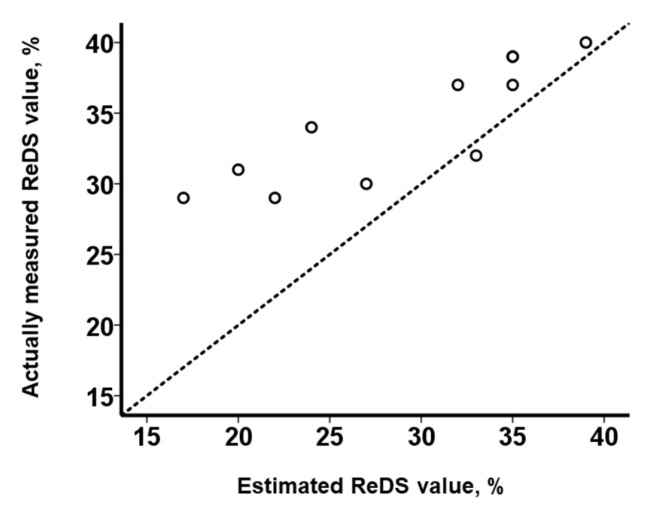
Relationship between actually measured ReDS values and the estimated ReDS values.

The linear regression equation in the PE group is as follows: (ReDS value (%)) = 0.47 × (PAWP (mmHg)) + 26.0. The linear regression equation in the no-PE group is as follows: (ReDS value (%)) = 0.73 × (PAWP (mmHg)) + 15.1. According to the linear regression equation of the no-PE group, the ReDS values under normal conditions without PE could be estimated. All actually measured ReDS values in the PE group were higher than the estimated ReDS values, except for one patient (34% (IQR: 31%, 38%) versus 30% (IQR: 23%, 35%, *p* = 0.005 according to Wilcoxon’s signed-rank test; Figure 6). In other words, ReDS values were more overestimated than expected in the presence of PE.

The ReDS values were estimated from the linear regression equation that was derived from the no-PE group: (ReDS (%)) = (PAWP (mmHg) + 12.3)/1.03. The dotted line represents the following: (actually measured ReDS value (%)) = (estimated ReDS value (%)).

All actually measured ReDS values were higher than the estimated ReDS values, except for one patient (*p* = 0.005 according to Wilcoxon’s signed-rank test). In other words, the actually measured ReDS values were overestimated in the presence of PE. The impact of the presence of PE on the ReDS values was significant, with a beta value of 6.61 (95% confidence interval: 4.80–8.42, *p* < 0.001) upon adjusting for the levels of PAWP. 

## 4. Discussion

In this study, we evaluated the effect of the presence of PE on the relationship between ReDS values and PAWP. In all cohorts, there was a moderate correlation between ReDS values and PAWP, as expected. PAWP was not significantly different between the PE group and the no-PE group, whereas ReDS values were significantly higher in the PE group than in the no-PE group. The ReDS values were more overestimated in the PE group than expected.

### 4.1. ReDS System to Estimate PAWP Levels

Right heart catheterization is the gold standard for assessing the severity of pulmonary congestion by measuring PAWP [4]. However, right heart catheterization is invasive, painful, and carries a risk of worsening heart failure, especially in patients with hemodynamic instability or excessive pulmonary congestion, or those receiving anticoagulants [3]. The indication of right heart catheterization is strictly restricted in daily clinical practice, and it is sometimes challenging to accurately evaluate the degree of pulmonary congestion given the lack of actually measured hemodynamics data.

The ReDS system has recently been introduced to assess the degree of pulmonary congestion [15]. This technology is non-invasive, easy and quick to measure, and does not require expert technicians. In the literature to date, various validation studies have been conducted that compare the ReDS system with other modalities that are clinically applied to assess pulmonary congestion, including chest computed tomography, right heart catheterization, lung ultrasound, chest X-ray, and plasma B-type natriuretic peptide levels [6,7,8,9,10,11,12]. ReDS values and PAWP are known to be moderately correlated [7,12], as also confirmed in this study. However, several confounders appear to affect ReDS values and may mislead clinicians in estimating pulmonary congestion by referencing ReDS values alone.

### 4.2. Impact of the Presence of PE on the Relationship between ReDS Values and PAWP

ReDS values are affected by several confounders. The ReDS system quantifies the intra-thoracic fluid amount that is located between two device sensors. If the amount of air increases (for example, chronic obstructive pulmonary disease or breath-holding in a maximum inspiratory state), the ReDS value will be underestimated [13,17]. We did not include such a pulmonary disease, and the ReDS values were measured under natural breathing conditions.

The presence of excessive extra-thoracic subcutaneous fat would inappropriately increase ReDS values [15]. The manufacturer does not recommend taking ReDS measurements in patients with body mass indexes over 35. We did not include such obese patients. The presence of serious lung diseases, including pulmonary pneumonia and lung cancer, can theoretically cause inappropriately elevated ReDS values [15]. We did not include those with such diseases.

Theoretically, another disease that may inappropriately increase ReDS values is PE. PAWP was not significantly different between the patients with and without PE, whereas ReDS values were inappropriately elevated in patients with PE compared with those without PE. The actually measured ReDS values in the PE group were higher than expected (the ReDS values were estimated from the data of patients without PE as a control).

It is no surprise that the ReDS system cannot distinguish extra-lung fluid (i.e., PE) from intra-lung fluid (i.e., pulmonary congestion). When patients have PE, we should pay special attention to estimating the degree of pulmonary congestion by using the ReDS system alone; ReDS values may be inappropriately elevated and the severity of pulmonary congestion may be overestimated. Other multiple modalities may be recommended to accurately interpret the severity of pulmonary congestion in patients with PE, instead of the ReDS system alone.

### 4.3. Limitations

This is a proof-of-concept study with a small sample size (the achieved power was calculated as 1.0 given an alpha value of 0.05 and an effect size of 0.813). We included patients who had received right heart catheterization. This is generally performed under relatively stable clinical conditions to avoid hemodynamic deterioration during/after the procedure [4]. Thus, we did not include patients with unstable hemodynamics with severe pulmonary congestion. The applicability of our findings to patients with more advanced heart failure with severe pulmonary congestion requires further studies. In patients with severe pulmonary congestion, its severity would be obvious, and detailed and accurate ReDS measurement may not necessarily be necessary. The recent literature proposes the aggressive surveillance of subclinical pulmonary congestion, even in patients without any signs of heart failure, given its negative prognostic impact [2]. Thus, we also included patients without heart failure.

The ReDS values appeared to be overestimated approximately 5% more than expected in patients with PE. The clinical implication of such a degree of overestimation of ReDS values may vary depending on each clinical scenario and requires further validation studies. For example, such an overestimation of ReDS values could have great implications in patients with relatively higher ReDS values.

All participants in the PE group had mild PE. The amount was between the first interquartile and the second interquartile lines. We could not evaluate the impact of severe PE, which may have a greater impact on ReDS values compared with mild PE. Right heart catheterization in such patients is challenging and has the risk of hemodynamic deterioration. Also, none of the patients with PE had obvious causes for their PE, such as malignancy or pneumonia. In other words, all PE cases in this study were idiopathic. The impact of PE on ReDS values may vary in each etiology.

## 5. Conclusions

In patients with PE, ReDS values were inappropriately elevated. The severity of pulmonary congestion may be overestimated when we use the ReDS system in patients with PE. Other modalities, such as right heart catheterization, are recommended for assessing the severity of pulmonary congestion in patients with PE.

## Figures and Tables

**Table 1:** Baseline characteristics.

	Total (*N* = 59)	PE (*N* = 11)	No PE (*N* = 48)	*p* Value
Demographics				
Age, years	79 (72, 84)	74 (70, 84)	79 (72, 84)	0.78
Male sex	30 (51%)	6 (55%)	24 (50%)	0.53
Body mass index	21.6 (19.5, 24.3)	22.5 (20.2, 23.6)	21.4 (19.3, 24.6)	0.44
Comorbidity				
Hypertension	49 (83%)	11 (100%)	38 (79%)	0.097
Dyslipidemia	26 (44%)	6 (55%)	20 (42%)	0.44
Diabetes mellitus	15 (25%)	4 (36%)	11 (23%)	0.36
Heart failure	31 (53%)	6 (55%)	25 (52%)	0.58
Atrial fibrillation	27 (46%)	4 (36%)	23 (48%)	0.36
History of stroke	5 (9%)	3 (27%)	2 (4%)	0.013 *
History of coronary intervention	6 (10%)	1 (9%)	5 (10%)	0.90
Echocardiography				
Left atrial diameter, mm	45 (38, 49)	48 (39, 59)	45 (38, 49)	0.18
LVDd, mm	48 (44, 57)	49 (46, 58)	48 (44, 57)	0.69
LVEF, %	54 (40, 69)	45 (28, 60)	57 (44, 71)	0.17
Moderate or grater MR	25 (42%)	3 (27%)	22 (46%)	0.26
Moderate or greater TR	18 (31%)	3 (27%)	15 (31%)	0.80
Hemodynamics				
Mean right atrial pressure, mmHg	8 (5, 10)	9 (7, 16)	6 (5, 10)	0.20
Mean pulmonary artery pressure, mmHg	21 (17, 26)	30 (21, 36)	21 (17, 24)	0.067
PAWP, mmHg	13 (10, 18)	21 (11, 24)	12 (10, 16)	0.079
Cardiac index, L/min/m^2^	2.4 (2.1, 2.9)	2.6 (1.7, 3.1)	2.4 (2.2, 2.8)	0.87
Systolic blood pressure, mmHg	108 (90, 131)	91 (81, 131)	108 (92, 130)	0.39
Heart rate, bpm	70 (63, 80)	72 (58, 91)	70 (64, 80)	0.39
Laboratory data				
Plasma BNP, pg/mL	231 (82, 474)	447 (245, 697)	198 (65, 454)	0.027 *
Medications				
Loop diuretics	33 (56%)	8 (73%)	25 (52%)	0.21
Beta-blocker	34 (58%)	6 (54%)	28 (58%)	0.82
Renin–angiotensin system inhibitor	46 (78%)	11 (100%)	35 (73%)	0.051
Mineralocorticoid receptor antagonist	28 (48%)	5 (45%)	23 (48%)	0.58
ReDS value, %	25 (22, 30)	34 (31, 38)	25 (22, 27)	<0.001 *

PE, pleural effusion; LVDd, left ventricular end-diastolic diameter; LVEF, left ventricular ejection fraction; MR, mitral regurgitation; TR, tricuspid regurgitation; PAWP, pulmonary artery wedge pressure; BNP, B-type natriuretic peptide; ReDS, remote dielectric sensing. Continuous variables are stated as median and interquartile range and were compared between the two groups using Mann–Whitney U test. Categorical variables are stated as numbers and percentages and were compared between the two groups using Fischer’s exact test. * *p* < 0.05.

## Data Availability

Data are available upon reasonable request from the corresponding author.
